# Supplementary material for: Isolation of Carboxylic Acids and NaOH from Kraft Black Liquor with a Membrane-Based Process Sequence
Source: Membranes (Basel). 2023 Jan 10;13(1):92. doi: 10.3390/membranes13010092 (PMC9863791; doi:10.3390/membranes13010092)
Supplement: Supplementary file 1 [file membranes-13-00092-s001.zip › membranes-2124619-supplementary.pdf]

membranes

Supporting Information for

Isolation of Carboxylic Acids and NaOH from Kraft Black Liquor with a Membrane-Based Process Sequence

Silvia Maitz, Lukas Wernsperger and Marlene Kienberger *

Institute of Chemical Engineering and Environmental Technology, Graz University of Technology, Inffeldgasse 25c, 8010 Graz, Austria
* Correspondence: marlene.kienberger@tugraz.at

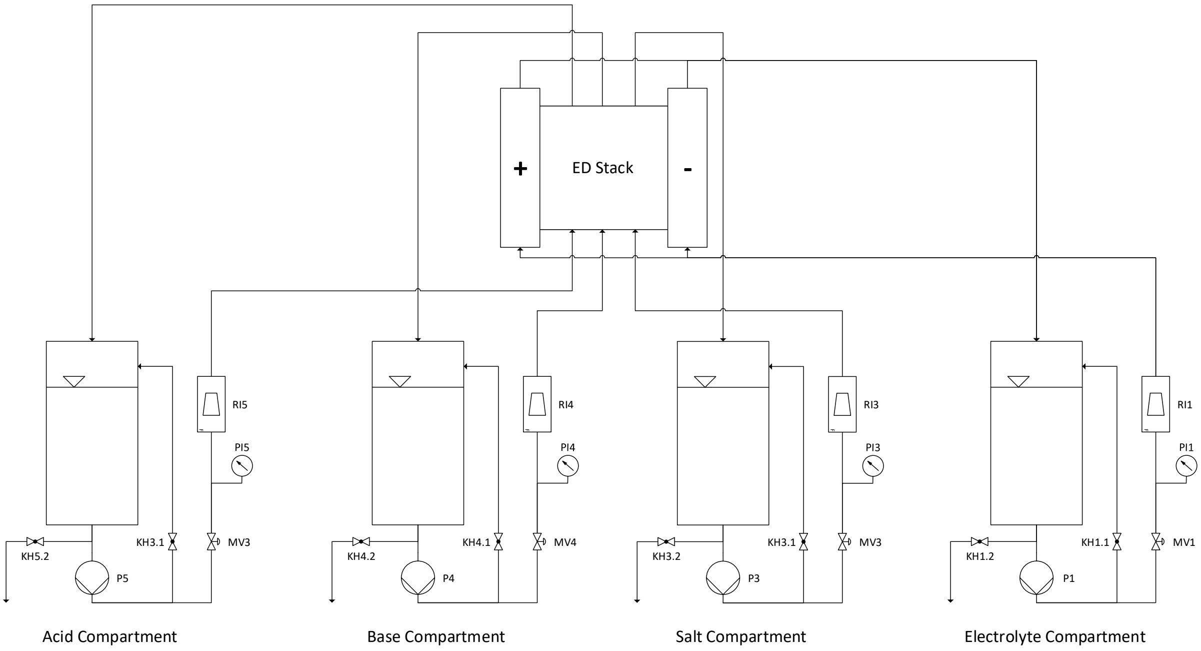

Figure S1. Set-up of the laboratory scale electrodialysis system.

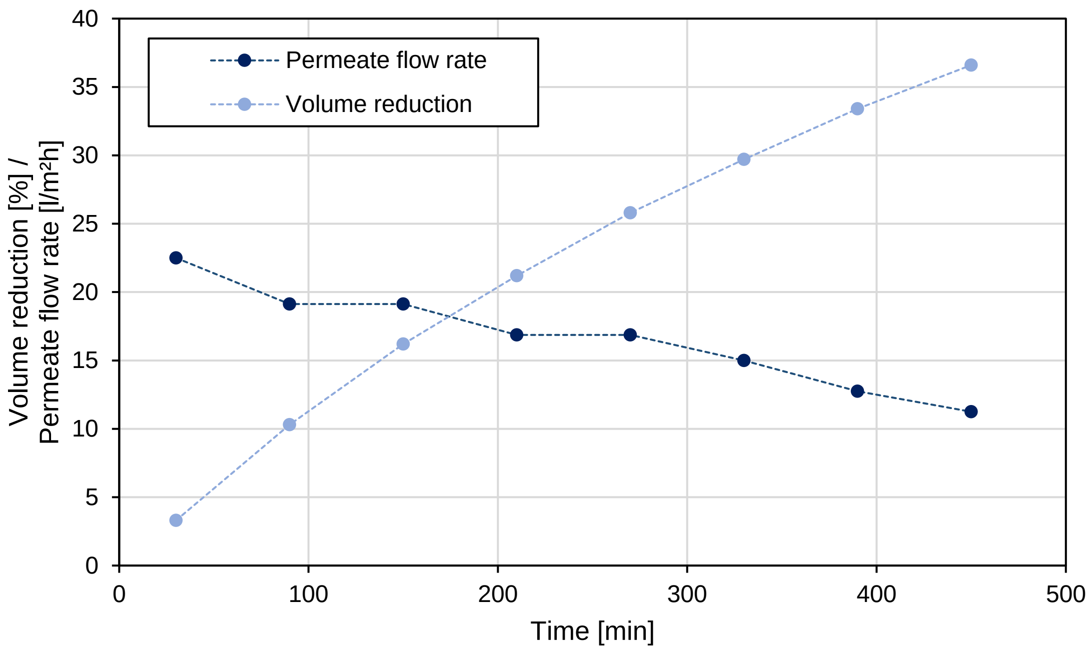

Figure S2. Development of permeate flow rate and volume reduction during a typical NF treatment of pre-treated kraft BL. Transmembrane pressure: 32 bar; temperature: 60 °C.

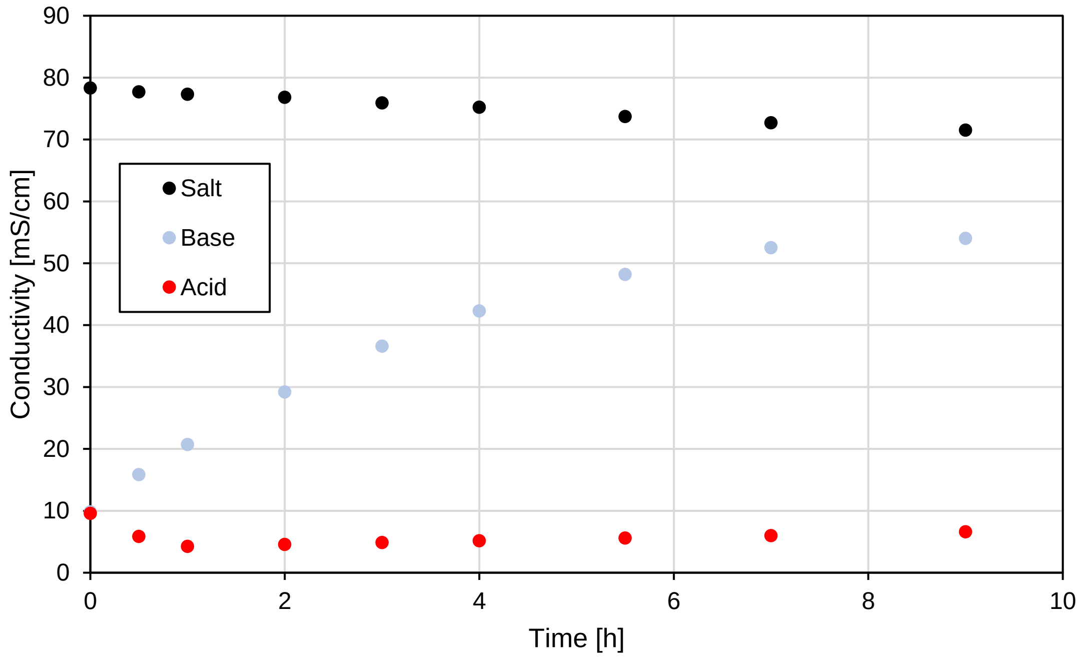

Figure S3. Development of the conductivity in the three different compartments during EDBM treatment of BL permeate.
